# A multilevel intervention to increase physical activity and improve healthy eating and physical literacy among young children (ages 3-5) attending early childcare centres: the Healthy Start-Départ Santé cluster randomised controlled trial study protocol

**DOI:** 10.1186/s12889-016-2973-5

**Published:** 2016-04-12

**Authors:** Mathieu Bélanger, Louise Humbert, Hassan Vatanparast, Stéphanie Ward, Nazeem Muhajarine, Amanda Froehlich Chow, Rachel Engler-Stringer, Denise Donovan, Natalie Carrier, Anne Leis

**Affiliations:** Department of family medicine, Université de Sherbrooke, 18 avenue Antonine-Maillet, Moncton, NB E1A 3E9 Canada; Centre de formation médicale du Nouveau-Brunswick, 18 avenue Antonine-Maillet, Moncton, NB E1A 3E9 Canada; Vitalité Health Network, 330 Université Avenue, Moncton, NB E1C 2Z3 Canada; College of Kinesiology, University of Saskatchewan, 7 Campus Drive, Saskatoon, SK S7N 5B2, Canada; College of Pharmacy and Nutrition /School of Public Health, University of Saskatchewan, 104 Clinic Place, Saskatoon, SK S7N 0Z2 Canada; Department of Community Health & Epidemiology, College of Medicine, University of Saskatchewan Health Sciences E Wing, 104 Clinic Place, Saskatoon, SK S7N 2Z4 Canada; École des sciences des aliments, de nutrition et d’études familiales, Faculté des sciences de la santé et des services communautaires, Université de Moncton, 18 avenue Antonine-Maillet, Moncton, NB E1A 3E9 Canada

**Keywords:** Childhood obesity, physical activity, physical literacy, food intake, eating habits, preschool, population health intervention

## Abstract

**Background:**

Childhood obesity is a growing concern for public health. Given a majority of children in many countries spend approximately 30 h per week in early childcare centers, this environment represents a promising setting for implementing strategies to foster healthy behaviours for preventing and controlling childhood obesity. Healthy Start-Départ Santé was designed to promote physical activity, physical literacy, and healthy eating among preschoolers. The objectives of this study are to assess the effectiveness of the Healthy Start-Départ Santé intervention in improving physical activity levels, physical literacy, and healthy eating among preschoolers attending early childcare centers.

**Methods/Design:**

This study follows a cluster randomized controlled trial design in which the childcare centers are randomly assigned to receive the intervention or serve as usual care controls. The Healthy Start-Départ Santé intervention is comprised of interlinked components aiming to enable families and educators to integrate physical activity and healthy eating in the daily lives of young children by influencing factors at the intrapersonal, interpersonal, organizational, community, physical environment and policy levels. The intervention period, spanning 6-8 months, is preceded and followed by data collections. Participants are recruited from 61 childcare centers in two Canadian provinces, New Brunswick and Saskatchewan. Centers eligible for this study have to prepare and provide meals for lunch and have at least 20 children between the ages of 3 and 5. Centers are excluded if they have previously received a physical activity or nutrition promoting intervention. Eligible centers are stratified by province, geographical location (urban or rural) and language (English or French), then recruited and randomized using a one to one protocol for each stratum. Data collection is ongoing. The primary study outcomes are assessed using accelerometers (physical activity levels), the Test of Gross Motor Development-II (physical literacy), and digital photography-assisted weighted plate waste (food intake).

**Discussion:**

The multifaceted approach of Healthy Start-Départ Santé positions it well to improve the physical literacy and both dietary and physical activity behaviors of children attending early childcare centers. The results of this study will be of relevance given the overwhelming prevalence of overweight and obesity in children worldwide.

**Trial registration:**

NCT02375490 (ClinicalTrials.gov registry).

## Background

Childhood obesity is one of the greatest challenges facing public health in the 21^st^ century [1–3]. Weight in early childhood closely predicts weight in later childhood [4–6] and children under six who are obese are at four times more at risk of becoming obese adults than normal weight children [7, 8]. Further, being overweight during childhood increases the likelihood to have compromised emotional, psychological and social well-being [9].

Many factors come together to produce obesity, but it is primarily due to an imbalance between energy intake and energy expenditure [10]. Eating habits are established early in childhood and usually persist for many years [11]. Data show that only 29 % of Canadian preschool aged children meet recommendations for fruit and vegetable intake and 23 % for grain products [12]. Further, 79 % of 4-5 year olds consume food of little nutritional value (e.g., chips, french fries, candy, chocolate, soft drinks, cake and cookies) at least once a week [12] and other studies have demonstrated that empty calories are making up as much as 40 % of their total caloric intake [13, 14]. Similarly, a recent review found that children in early childcare centers (ECC) have low levels of physical activity, and that they are sedentary for much of the time [15]. It was estimated that children in childcare settings accumulate an average of only 7 to 13 min of moderate to vigorous physical activity during the course of a 7 h day [16]. Moreover, recent data show that many of them have poor physical literacy [17–21]. These data are troubling given that sedentary state and physical activity levels track over time [22–26].

Interventions designed to improve the physical activity and nutrition of preschoolers are needed to prevent and control childhood obesity [1, 27–29]. Since more than half of Canadian preschoolers spend an average of 29 h a week in ECC [30], this environment is a prime setting for implementing an array of strategies to foster healthy behaviours [31–36]. However, two systematic reviews on obesity prevention in children under 5 years, one on interventions [35] the other on preventive policies, practice and interventions in ECC [36], reported limited success in improving physical activity levels, dietary behaviour, or body composition. The authors suggest that the least successful interventions focused only on one or two outcomes, while the most successful interventions positively influenced factors such as knowledge, abilities and competence. According to the authors, this means that interventions should be grounded in comprehensive behaviour change models, be multifaceted and sustained over time [35]. Few interventions focused on physical activity and eating behaviours simultaneously, therefore future interventions should target both behaviours [36]. It can be concluded that interventions promoting healthy weights in children should encompass a broad spectrum of concerted actions and be based on best available knowledge from research and practice [16, 37].

Healthy Start-Départ Santé, a multilevel intervention that targets physical activity, physical literacy, and healthy eating in preschoolers, was developed following these principles. The aim of the current study is to lead a comprehensive evaluation of the Healthy Start-Départ Santé intervention using an experimental research design.

### The intervention

Healthy Start-Départ Santé uses a population health approach to promoting physical activity and healthy eating among English- and French-speaking preschoolers between 3–5 years old who attend ECCs (e.g., licenced childcares or preschools). The population health approach posits that to positively influence population-level health outcomes, interventions must take into account the wide range of health determinants [38], recognise the importance and complexity of potential interplay among these determinants, and reduce social and material inequities [39]. Further, they must rely on best evidence available, stimulate intersectoral collaborations, and provide opportunities for all potential stakeholders to be meaningfully engaged from the onset to their deployment [39]. Several models based on the population health approach have been developed to steer interventions [40–42] and, similar to ecological models [43], they call for interventions to include a series of concerted actions capable of targeting all levels of influence such as the intrapersonal (biological and psychological), interpersonal (social and cultural), organizational, community, physical environment and political levels. Healthy Start-Départ Santé includes strategies for each level of influence. Supported by local funding, and then by Phase I of the Public Health Agency of Canada Innovation Strategy (2008-2012), Healthy Start-Départ Santé was developed by researchers, community groups, educators, parents, and government representatives, pilot tested, and adapted to diverse contexts. In particular, Healthy Start-Départ Santé was linguistically and culturally adapted to cater to both official linguistic groups in Canada, which is important since it has been documented that to be effective, interventions must be tailored and implemented using several different socio-linguistic perspectives as befitting the target population [44].

**Fig. 1:**
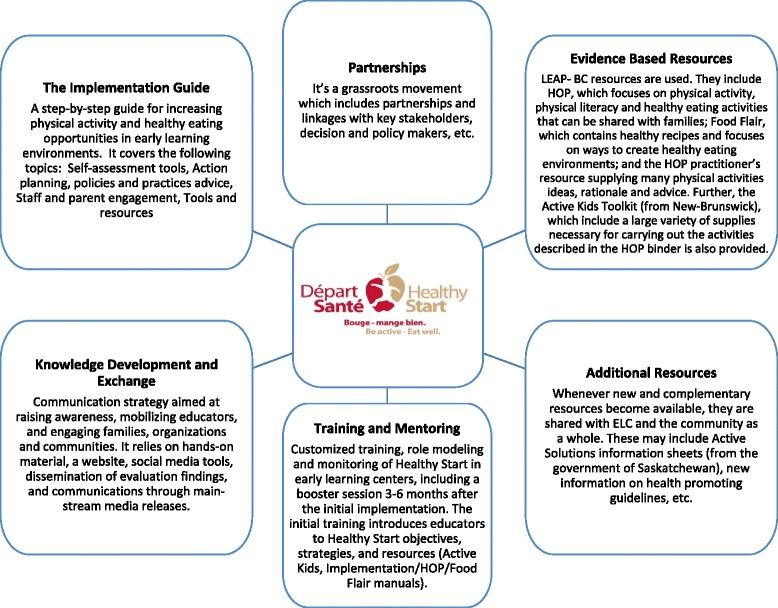
Healthy Start components

The mission of Healthy Start-Départ Santé is to encourage and enable families and educators to integrate physical activity and healthy eating in the daily lives of young children. Specifically, Healthy Start-Départ Santé attempts to influence factors at the intrapersonal (e.g., eating and physical activity behaviour of children), interpersonal (e.g., educators and parents), organizational (e.g., ECC), community (e.g., community organization involvement), physical environment and political levels (e.g., built environment and policies). The intervention is composed of six interlinked components which are presented in more detail in Fig. 1. These components include: 1) intersectoral partnerships conducive to participatory action that leads to promoting healthy weights in communities and ECC; 2) the Healthy Start-Départ Santé implementation manual for educators on how to integrate healthy eating and physical activity in their centre; 3) customized training, role modelling and monitoring of Healthy Start-Départ Santé in ECC; 4) the evidence-based resource, LEAP-GRANDIR [16], which contains material for both families and educators; 5) supplementary resources from governmental partners; and 6) a knowledge development and exchange (KDE), and communication strategy involving social media and web-resources to raise awareness and mobilize grassroots organizations and communities. Healthy Start-Départ Santé is delivered over 6-8 months and includes a partnership agreement, an initial training session which orients ECC staff to the concepts, the implementation manual and the use of resources, on-going support and monitoring over time, one tailored booster session, and a family day to celebrate the ECC’ success at the end of the intervention.

### Study objectives

It is hypothesized that, in comparison to usual practice, exposure to the Healthy Start-Départ Santé intervention will lead to improved opportunities for physical activity and healthy eating hence to increased physical activity and healthier eating among children. The specific study objectives are to assess whether:

1. The Healthy Start-Départ Santé intervention leads to increases in opportunities for physical activity and healthy eating in ECC through improved knowledge, attitudes and self-efficacy of the educators and directors;
2. The Healthy Start-Départ Santé intervention leads to increases in physical activity levels and healthy eating behaviors among preschoolers, in turn promoting healthy weights;
3. The Healthy Start-Départ Santé intervention leads to improvements in physical literacy among preschoolers.

## Methods/Design

### Study design

**Fig. 2:**
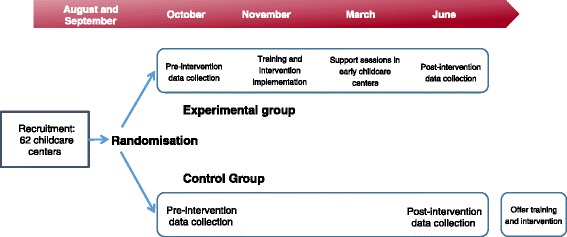
Data collection timeline for the Healthy Start study

The Healthy Start-Départ Santé evaluation follows a delayed cluster randomized controlled trial design in which the ECCs are randomly assigned to receive the intervention or serve as usual practice controls. The intervention spans a period of 6–8 months. This period is both preceded and followed by data collections (Fig. 2). Control sites are given the option of receiving the intervention once their participation in the evaluation has been completed. Data collection takes place over three years, such that approximately a third of ECCs recruited will complete the study each year. This study protocol received ethics approval from Health Canada, the University of Saskatchewan, and the Université de Sherbrooke.

### Target population and sampling

Participants for the Healthy Start-Départ Santé project are recruited from 61 French and English ECCs in two Canadian provinces: New Brunswick and Saskatchewan. Province-specific recruitment and data collection are carried through a study coordinating center established in each province. Both coordinating centers follow the same protocols. Specifically, a registry of all licenced ECCs in both provinces is obtained. From this list we exclude any ECC which has already received a physical activity or nutrition promoting intervention in the past. This avoids underestimating the effect of the Healthy Start-Départ Santé intervention. To be included in the study, an ECC has to prepare and provide meals for lunch. This is required for assessing the quality of foods being served and for measuring nutritional intake. For feasibility and efficiency reasons, the number of children attending the ECC also serves as an exclusion criterion; centers with less than 20 children between the ages of 3 and 5 are not considered. Information on these criteria is obtained from governmental partners or through a brief telephone administered questionnaire. In order to obtain a final sample of at least 735 children, we estimated that a minimum of 15 children aged 3 to 5 would need to be recruited in 62 ECCs. A minimum of 20 children per center was established to account for a 60 % participation rate (Recruiting 60 % of eligible children in 62 ECCs would give a total of approximately 744 participants). Despite planned measures to minimize losses to follow-up, this issue is inevitable in a longitudinal study of this nature. Based on pilot work, we anticipate an attrition rate of approximately 5 %, which will provide adequate statistical power to conduct the planned analyses. The target of 735 children was based on estimates that 700 (735 - 5 %) participants divided into two groups of 350 participants will provide 80 % power to detect a 10 % between-group difference in outcome considering a within-group standard deviation of 40 %, α = 0.05, intra-class correlation of 0.02, and collinearity between the intervention and other explanatory variables expressed by an estimated multiple correlation of 0.15.

**Fig. 3:**
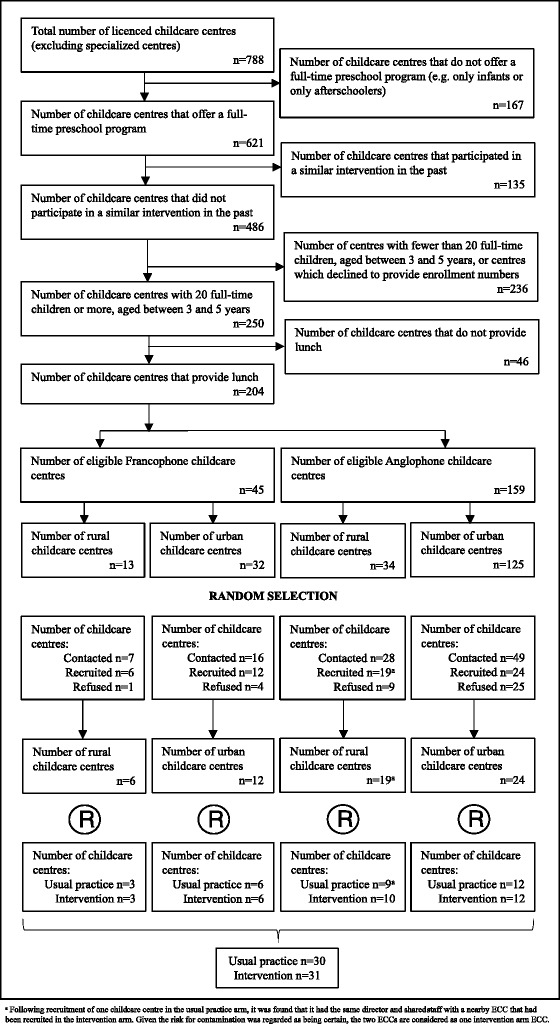
Flow diagram of selection and randomisation process for the Healthy Start study.

For recruiting purposes, the ECCs are stratified according to province, geographical location (urban and rural) [45] and their respective school district (English or French) (Fig. 3). This stratification process ensures relatively homogenous strata for comparisons, since curriculum can differ between ECCs under different district authorities and that the ECC environment can be influenced by its geographical location [46, 47]. Once stratification is completed, eligible ECCs are randomly selected through a sequence generated using Stata SE statistical software (StataCorp, College Station, TX) according to the above-mentioned strata. Given the large area Saskatchewan represents (652 000 km^2^), it was decided to carry the study with selected ECCs in the central region in year 1, and in the South and central-North in years 2 and 3. Selected ECC are contacted, provided with information, and invited to participate in the project. Subsequently, ECC directors are telephoned to answer their questions and to confirm their participation while securing the parents’ board support. The recruited ECC are then sent consent forms. Centres which decline participation or with fewer than five children recruited are replaced by other randomly selected ECC from the same stratum. Once the ECC provides final consent, it is randomly allocated to the intervention or usual practice (control) arm. A one to one randomization protocol is applied to each stratum. Invitation packages describing the study and seeking parental consent are sent to parents of all age-eligible children. ECCs return completed parental consent forms to the provincial coordinating centres. Following recruitment of one ECC and its children in the usual practice arm, it was found that it had the same director and shared staff with a nearby ECC that had been recruited in the intervention arm. Given the risk for contamination was regarded as being certain in this situation, the two ECC are considered as one intervention arm ECC, which explains the final sample of 61 ECC enrolled in the study.

### Main outcome measures

To assess the objectives specified earlier, each of the measures below is administered to participants in the intervention group before and after the intervention period. The measures are administered at the same times to participants in the control group.

### Physical activity level

Children’s physical activity levels are obtained using an Actical accelerometer worn during attendance of ECC for five consecutive weekdays. Accelerometers represent an objective and valid method of measuring physical activity in preschoolers [48–50]. The Actical, an omnidirectional accelerometer, was demonstrated to have higher intra- and inter-instrument reliability than other accelerometers [51]. It correlates at *r* = 0.89 with directly measured oxygen consumption [50]. For this study, accelerometer data are recorded in 15 s intervals. Data will be cleaned and managed using the procedure recommended by Statistic Canada [52, 53] through a series of publicly available SAS codes adapted for this type of study [54].

Educators’ physical activity levels are measured with pedometers (New Lifestyles SW-200 DIGI-WALKER). The SW-200 pedometers, commonly used in applied research, have consistently shown to be among the most accurate at counting steps in controlled laboratory settings [55, 56], and have demonstrated acceptable reliability in real-life settings [57]. Educators are given a log in which to record the number of steps taken each day during work at the ECC for the same five consecutive weekdays.

### Physical literacy

Physical literacy and gross motor skills of children are measured using the Test of Gross Motor Development (TGMD-II). The TGMD-II is a standardized test designed to assess the gross motor functioning of children aged 3 through 10 years [58]. It evaluates two subtests of skills: locomotor (run, hop, gallop, leap, horizontal jump and slide) and object control (ball skills such as striking a stationary ball, stationary dribble, catch, kick, overhand throw, and underhand roll). Of the 12 skills included in the TGMD-II, three (slide, striking with a stationary ball and stationary dribble) are not assessed as they were found particularly difficult and rarely performed adequately by children under six years in pilot work. This was decided in concertation with developers of the TGMD-II. Each child is videotaped while completing the skills. Videos are then reviewed by trained researchers who score two trials for each subtest. Moderate to strong correlations were reported between the TGMD-II and the Comprehensive Scales of Student Abilities, and an inter-rater correlation coefficient of 0.98 indicate good test-retest reliability [58].

### Quality of food served to children

Menus and recipes for mixed dishes are collected from each ECC. From these, the types and amounts of meals and snacks served are recorded. Information on food provided is standardized in terms of category and food group as well as number and portion size served.

### Food consumed by children

Food intake in ECCs is assessed using the digital photography-weighted plate waste method on two consecutive days. Food consumed is measured by weighing each food served to participants with a digital scale that was manually calibrated (inStyle, 16 × 22 × 1.5 cm, Home Hardware Stores Limited, Canada, 2009) and simultaneously taking a digital picture of the food, and the plate ware on which it was served, with an “ASUS Memo Pad HD7” (ME173X model, Android US, China, 2013). Any leftovers of those foods are then weighed and pictured again at the end of the meal. This is done for every serving. Pictures are taken at a distance of 62 cm and at a 45° angle [59]. This method has been extensively used in studies of school-aged children [60–62] and is considered the most precise measurement of dietary intake [63, 64]. Digital photography has recently been used to characterize meals served and consumed at pre-schools and it is correlated at 0.92 (*p* < 0.0001) with the weighing method [59]. Information on amount of calories, various food groups, macronutrients, vitamins and minerals consumed is derived by subtracting the weight of leftover of a given food from the weight served. For foods containing multiple food groups, trained research assistants used the pictures to estimate the proportion of food group consumed. This step is assisted by the Food Processor nutritional analysis software (Food Processor, Esha version 10.10.00).

### Eating habits and nutritional risks

The Nutrition Screening Tool for Every Preschooler (NutriSTEP), designed and validated in both English and French for 3–5 year-old Canadian children of different ethnicities, is used to assess eating habits and nutrition problems in children [65]. This screening tool consists of 17 questions and takes parents or primary caregivers about five minutes to complete. Assessment of nutritional risk using the NutriSTEP has been validated against registered dietitians’ nutrition assessments (*r* = 0.49, *p* = 0.01) [66]. Intraclass correlation of 0.89 (*p* < 0.001) indicates good test-retest reliability and the majority of items have a Kappa percent of agreement ranging from adequate (κ > 0.5) to excellent (κ > 0.75) [66]. NutriSTEP includes a validated semi-quantitative food frequency questionnaire to assess the usual intake of fast foods as well as the four main food groups according Canada’s Food Guide to Healthy Eating: fruits and vegetables, milk and alternatives, meat and alternatives, grain products. This enables us to estimate the contribution of meals consumed in ECCs to the total intake of the four main food groups.

### Early childcare centre environment

Dietary and physical activity practices and policies in ECC are measured using items from the Nutrition and Physical Activity Self-Assessment of Child Care (NAP SACC) [67–69]. The 55-items of the NAP SACC retained for this study collect information on 5 components of nutrition (25 items assessing: feeding environment, feeding practices, menus and variety, education and professional development, policy) and 5 components of physical activity (30 items assessing: time provided, indoor and outdoor play environment, educator practices, education and professional development, policy) [70]. Each item is attributed a score between 0 and 3 points. The NAP SACC is filled out independently by two research assistants in each ECC over the course of 2 consecutive days. Differences in item scores are discussed between research assistants, who then come to a consensus. NAP SACC showed excellent inter-rater reliability with 65 % of items having 100 % agreement and all other items having agreement scores exceeding 71 %.

*Knowledge, attitudes, practices and self-efficacy of educators and directors* is measured through a self-administered questionnaire. The educator questionnaire collects information on educator practices related to nutrition (12 items, e.g., role modelling healthy food choices) and physical activity (6 items, e.g., incorporating physical activity into classroom routines and transitions) [68], on their self-perceived knowledge of fundamental movement skills (4 items, e.g., level of knowledge of activities that incorporate motor skills in the daily activities of preschoolers), on their self-efficacy (e.g., rate your level of confidence in …) to promote physical activity (1 item), and to teach fundamental movement skills (1 item) [71, 72]. Educators also provide information on their physical activity level through the International Physical Activity Questionnaire-Short form [73], food choices based on an adapted version of the NutriStep, and sociodemographic information (including their age group, level of education, and the length of time that they have been working as an educator).

### Complementary measures or co-variables

Beyond the main measures which relate directly to the objectives, the following measures are also administered at the beginning and the end of the study to account for potentially confounding factors in the analyses and to assess secondary outcomes.

### Sociodemographic information

Parents provide sociodemographic information through a self-reported questionnaire. Questions in the questionnaire are drawn from Statistic Canada’s Canadian Community Health Survey [74]. The questionnaire provides information on parents’ education levels (highest level of education completed by each parent), family structure (number of siblings and family status) and socioeconomic level (estimate of total family revenues, before deductions, in the last 12 months). Parents also report their physical activity levels and food choices based on the same questions as the educator questionnaire. Parents’ perception of their child’s physical activity abilities are assessed through 4 items of the Perceptions of Physical Activity Importance and their Children’s Ability Questionnaire (PPPAICAQ) [75]. The NutriStep questionnaire is also completed by parents.

### Body composition

Height, weight and waist circumference of children are measured using a standardized protocol [76]. Two measures of height (SECA Stadiometer – Model 213) to the nearest 0.1 cm, weight (SECA Scale – Model 761) to the nearest 0.2 kg, and waist circumference to the nearest 0.1 cm are obtained for each participant. If discrepancies greater than 0.5 cm for height and waist circumference or 0.2 kg for weight are observed between the two measures, a third measure is obtained. The average of the two closest measures is recorded. Body mass index (BMI), calculated using the ratio of weight (kg) and squared height (m^2^) will be used to determine if children are overweight or obese by following the International Obesity Task Force age-adjusted thresholds as recommended [77].

### Training of research assistants

Before every data collection periods, research assistants are given a full day of training on data collection and entry to ensure that all procedures are standardized across sites. Research assistants are trained specifically on how to collect anthropometric data, how to conduct plate waste analysis, and how to complete the environmental scan. They also practice all fundamental movement patterns so that they can demonstrate them correctly, and review how to properly videotape children’s movements. Databases are also presented, and research assistants are trained on how to enter all data collected. Provincial study coordinators oversee all aspects of data collection and data entry to ensure protocols are applied as intended.

### Data analysis

As a first step, each variable distribution will be examined in order to manage obvious errors such as outliers or questionable input. If necessary, appropriate transformations will be applied. If there are systematic missing variables, these will be filled using multiple imputation methods. After the initial data cleaning and processing, descriptive analyses will be used to characterise the two groups of participants and their context.

Analyses of the main study objectives will adhere to the intention-to-treat principle. Multiple linear regression will be used to determine if, in comparison to participants in the usual care group, participants exposed to Healthy Start-Départ Santé had greater increases in ECC-provided opportunities for physical activity, ECC-provided opportunities for healthy eating, in physical activity levels, in healthy eating behaviours, and if they had greater improvements in physical literacy. A multilevel approach will be used in each model to account for clustering that could result from children attending the same ECC. Potentially confounding variables (i.e., age, parental education, household income, etc.) will be included in the models.

### Limitations

Educator turnover is common in ECC, meaning that new educators in centers offering the Healthy Start-Départ Santé intervention may not receive the intended training. Two elements of the intervention nevertheless reduce this possibility. First, one booster session per centre is offered as part of the intervention to reinforce the training of educators who are there since the beginning and to ensure that others are trained to implement Healthy Start-Départ Santé. Second, Healthy Start-Départ Santé is designed to be integrated into centres’ everyday functioning so that all employees should be exposed to it naturally. Also, children’s physical activity level and dietary intake can vary from one day to the next. This could cause the data to be non-representative of typical behavior. Data collection over five consecutive days for physical activity and two consecutive days for dietary behavior will help minimise this risk.

### Trial status

Participant recruitment began in autumn 2013. Intervention delivery and data collection began at the same time and are ongoing. This trial is registered through the ClinicalTrials.gov registry (Clinical Trials ID = NCT02375490; Protocol ID = 6282-15-2010/3381056-RSFS).

## Discussion

Framed within the population health approach and based on six interlinked components, the Healthy Start-Départ Santé study is positioned to improve the physical literacy and both dietary and physical activity behaviours of children attending ECC. The results of this study will be of particular relevance given the overwhelming prevalence of overweight and obesity in children worldwide, especially in North America. The current study has numerous strengths, including the use of a randomized control trial design, longitudinal follow up, objective measures of the main outcomes, and a population-based sample with sufficient subjects. In addition to a rigorous scientific approach, Healthy Start-Départ Santé is culturally adapted for the two official linguistic groups in Canada and targets multiple levels of influence. The fact that it is based on strong multi-sectorial collaborations with a variety of stakeholders improves its feasibility and potential of its wide-spread implementation post-evaluation.

To date, few intervention studies have applied multifaceted methods to obesity prevention in preschoolers. Healthy Start-Départ Santé is among the first to capitalize on a combination of multiple partner involvement and interventions on multiple socioecological levels with over 6 months of follow-up in order to affect two behaviours which are fundamental causes of obesity.

Healthy Start-Départ Santé is also well setup to enable epidemiological investigations. For example, we will use Healthy Start-Départ Santé data to quantify the relative importance of different components of the population health model in explaining different levels of involvement in the physical activity and the various dietary behaviors of preschoolers. Similarly, the data will enable investigating how different determinants may interact to influence behaviour change in young children, which to date has not been explored extensively. This combination of effectiveness testing and epidemiological analyses will allow gaining insights into processes and events that lead to behavioral changes in preschoolers. All of this will guide the refinement of the current intervention and the development of new ones in order to increase physical activity and improve dietary behavior and physical literacy among young children.

## Abbreviations

ECC: Early childcare centers
KDE: Knowledge development and exchange
NAP SACC: Nutrition and physical activity self-assessment of child care
NutriSTEP: Nutrition screening tool for every preschooler
TGMD-II: Test of gross motor development

## References

[1] WHO (2012). Population-based approaches to childhood obesity prevention.

[2] de Onis M, Blössner M, Borghi E (2010). Global prevalence and trends of overweight and obesity among preschool children. Am J Clin Nutr.

[3] Shields M (2006). Overweight and obesity among children and youth. Heal Reports.

[4] Nader PR, O’Brien M, Houts R, Bradley R, Belsky J, Crosnoe R, Friedman S, Mei Z, Susman EJ (2006). Identifying risk for obesity in early childhood. Pediatrics.

[5] Quattrin T, Liu E, Shaw N, Shine B, Chiang E (2005). Obese children who are referred to the pediatric endocrinologist: characteristics and outcome. Pediatrics.

[6] Gardner D, Hosking J, Metcalf B, Jeffery A, Voss L, Wilkin T (2009). Contribution of early weight gain to childhood overweight and metabolic health: a longitudinal study. Pediatrics.

[7] Guo S, Huang C, Maynard L, Demerath E, Towne B, Chumlea W, Siervogel R (2000). Body mass index during childhood, adolescence and young adulthood in relation to adult overweight and adiposity: the Fels longitudinal study. Int J Obes Relat Metab Disord.

[8] Freedman D, Kettel Khan L, Serdula M, Dietz W, Srinivasan S, Berenson G (2005). The relation of childhood BMI to adult adiposity: the Bogalusa Heart Study. Pediatrics.

[9] Reilly J, Methven E, McDowell Z, Hacking B, Alexander D, Stewart L, Kelnar C (2003). Health consequences of obesity. Arch Dis Child.

[10] Hill JO, Melanson EL (1999). Overview of the determinants of overweight and obesity: current evidence and research issues. Med Sci Sports Exerc.

[11] Birch L, Fisher J (1998). Development of eating behaviors among children and adolescents. Pediatrics.

[12] Pabayo R, Spence JC, Casey L, Storey K (2012). Food consumption patterns in preschool children. Can J Diet Pract Res.

[13] Reedy J, Krebs-Smith SM (2010). Dietary sources of energy, solid fats, and added sugars among children and adolescents in the United States. J Am Diet Assoc.

[14] Langlois K, Garriguet D (2011). Sugar consumption among Canadians of all ages. Heal Reports.

[15] Reilly J, Kelly J (2011). Long-term impact of overweight and obesity in childhood and adolescence on morbidity and premature mortality in adulthood: systematic review. Int J Obes.

[16] Temple V, Naylor P, Rhodes R, Higgins J (2009). Physical activity of children in family child care. Appl Physiol Nutr Metab.

[17] Hardy LL, Reinten-Reynolds T, Espinel P, Zask A, Okely AD (2012). Prevalence and correlates of low fundamental movement skill competency in children. Pediatrics.

[18] Fisher A, Reilly JJ, Kelly LA, Montgomery C, Williamson A, Paton JY, Grant S (2005). Fundamental movement skills and habitual physical activity in young children. Med Sci Sports Exerc.

[19] Gentier I, D’Hondt E, Shultz S, Deforche B, Augustijn M, Hoorne S, Verlaecke K, De Bourdeaudhuij I, Lenoir M (2013). Fine and gross motor skills differ between healthy-weight and obese children. Res Dev Disabil.

[20] Lubans DR, Morgan PJ, Cliff DP, Barnett LM, Okely AD (2010). Fundamental movement skills in children and adolescents: review of associated health benefits. Sports Med.

[21] Williams HG, Pfeiffer KA, O’Neill JR, Dowda M, McIver KL, Brown WH, Pate RR (2008). Motor skill performance and physical activity in preschool children. Obesity (Silver Spring).

[22] Kjønniksen L, Torsheim T, Wold B (2008). Tracking of leisure-time physical activity during adolescence and young adulthood: a 10-year longitudinal study. Int J Behav Nutr Phys Act.

[23] Telama R, Yang X, Viikari J, Valimaki I, Wanne O, Raitakari O (2005). Physical activity from childhood to adulthood: a 21-year tracking study. Am J Prev Med.

[24] Tammelin T (2005). A review of longitudinal studies on youth predictors of adulthood physical activity. Int J Adolesc Med Heal.

[25] Telama R (2009). Tracking of physical activity from childhood to adulthood: a review. Obes Facts.

[26] Hallal P, Victora C, Azevedo M, Wells J (2006). Adolescent physical activity and health: a systematic review. Sport Med.

[27] About Obesity in Canada - Canadian Obesity Network [http://www.obesitynetwork.ca/obesity-in-canada]

[28] Baur LA (2009). Tackling the epidemic of childhood obesity. CMAJ.

[29] Larson N, Ward DS, Neelon SB, Story M (2011). What role can child-care settings play in obesity prevention? A review of the evidence and call for research efforts. J Am Diet Assoc.

[30] Bushnik T (2006). Child Care in Canada.

[31] Dooris M, Poland B, Kolbe L (2007). Healthy Settings: Building evidence for the effectiveness of whole system health promotion. Challenges and future directions. Global Perspectives on Health Promotion Effectiveness.

[32] Sharma S, Chuang R, Hedberg A (2011). Pilot-testing CATCH early childhood: a preschool-based healthy nutrition and physical activity program. Am J Heal.

[33] Stettler N, Bhatia J, Parish A, Stallings V (2011). Feeding healthy infants, children, and adolescents. In Nelson Textbook of Pediatrics.

[34] Story M, Kaphingst K, French S (2006). The role of child care settings in obesity prevention. Futur Child.

[35] Hesketh KD, Campbell KJ (2010). Interventions to prevent obesity in 0–5 year olds: an updated systematic review of the literature. Obesity (Silver Spring).

[36] Kaphingst KM, Story M (2009). Child care as an untapped setting for obesity prevention: state child care licensing regulations related to nutrition, physical activity, and media use for preschool-aged children in the United States. Prev Chronic Dis.

[37] Public Health Agency of Canada and the Canadian Institute for Health Information. Obesity in Canada. Ottawa: Government of Canada; 2011.

[38] Evans R, Barer M, Marmor T (1994). Why Are Some People Healthy and Others Not?: The Determinants of Health of Populations.

[39] Implementing the Population Health Approach - Public Health Agency of Canada [http://www.phac-aspc.gc.ca/ph-sp/implement/index-eng.php]

[40] Kumanyika S, Jeffery RW, Morabia A, Ritenbaugh C, Antipatis VJ (2002). Obesity prevention: the case for action. Int J Obes Relat Metab Disord.

[41] Prince S (2009). A population health approach to obesity in Canada—Putting the problem back into context. Transdiscipl Stud Popul Heal Ser..

[42] Huynen MMTE, Martens P, Hilderink HBM (2005). The health impacts of globalization: a conceptual framework. Global Health.

[43] Sallis J, Owen N, Fisher E, Glanz K, Rimer BK, Viswanath K (2008). Ecological models of health behavior. Health Behavior and Health Education: Theory, Research, and Practice.

[44] Kao H, Hsu M, Clark L (2004). Conceptualizing and Critiquing Culture in Health Research. J Transcult Nurs.

[45] Community Information Database - Metropolitan Influence Zone (MIZ) Topology [http://map.cid-bdc.ca/#s=2006;i=comtype.miz;sly=can_sdr_DR;sid=689;v=map1;l=en;z=2149095,322536,269304,188240]

[46] Maher E, Frestedt B, Grace C (2008). Differences in Child Care Quality in Rural and Non-Rural Areas. J Res Rural Educ.

[47] Swenson K. Child care arrangements in urban and rural areas. US Dep Heal Hum Serv Pg. 2008; p. 1–16.

[48] Adolph A, Puyau M, Vohra F, Nicklas T, Zakeri I, Butte N (2012). Validation of uniaxial and triaxial accelerometers for the assessment of physical activity in preschool children. J Phys Act Heal.

[49] Pate RR, O’Neill JR, Mitchell J (2010). Measurement of physical activity in preschool children. Med Sci Sports Exerc.

[50] Pfeiffer K, Mciver K, Dowda M, Almeida M, Pate R (2006). Validation and calibration of the Actical accelerometer in preschool children. Med Sci Sports Exerc.

[51] Esliger D, Tremblay M (2006). Technical reliability assessment of three accelerometer models in a mechanical setup. Med Sci Sports Exerc.

[52] Colley R, Connor Gorber S, Tremblay M (2010). Quality control and data reduction procedures for accelerometry-derived measures of physical activity. Heal Reports.

[53] Colley R, Garriguet D, Janssen I, Craig C, Clarke J, Tremblay M (2011). Physical activity of Canadian children and youth: accelerometer results from the, to 2009 Canadian Health Measures Survey. Heal Reports.

[54] Boudreau J, Bélanger M. SAS Code for Actical Data Cleaning and Management: Version 1.3. Centre de formation médicale du Nouveau-Brunswick. Moncton; 2015. http://mathieubelanger.recherche.usherbrooke.ca/Files/Other/UserManual_BoudreauBelanger%20V1-3.pdf

[55] Crouter SE, Schneider PL, Karabulut M, Bassett DR (2003). Validity of 10 electronic pedometers for measuring steps, distance, and energy cost. Med Sci Sports Exerc.

[56] Schneider PL, Crouter SE, Lukajic O, Bassett DR (2003). Accuracy and reliability of 10 pedometers for measuring steps over a 400-m walk. Med Sci Sports Exerc.

[57] Schneider PL, Crouter SE, Bassett DR (2004). Pedometer measures of free-living physical activity: comparison of 13 models. Med Sci Sports Exerc.

[58] Ulrich D (2000). Test of gross motor development-2.

[59] Williamson DA, Allen HR, Martin PD, Alfonso AJ, Gerald B, Hunt A (2003). Comparison of digital photography to weighed and visual estimation of portion sizes. J Am Diet Assoc.

[60] Blakeway SF, Knickrehm ME (1978). Nutrition education in the Little Rock school lunch program. J Am Diet Assoc.

[61] Lee H, Lee K, Shanklin C (2001). Elementary students’ food consumption at lunch does not meet recommended dietary allowance for energy, iron, and vitamin A. J Am Diet.

[62] Whatley J, Donnelly J. Energy and macronutrient consumption of elementary school children served modified lower fat and sodium lunches or standard higher fat and sodium lunches. J Am Coll Nutr. 1996;15(6):602-7. 10.1080/07315724.1996.10718636 8951738

[63] Jacko C, Dellava J, Ensle K, Hoffman D. Use of the Plate-Waste method to Measure food intake in children. J Ext. 2007;45:6RIB7.

[64] Wolper C, Heshka S, Heymsfield S, Allison D (1995). Measuring food intake: An overview. Handbook of Assessment Measures for Eating Behaviors and Weight-Related Problems.

[65] NutriSTEP® - NutriSTEP® Implementation Toolkit and Resources [http://nutristep.ca/en/toolkit_resources.aspx]

[66] Randall Simpson JA, Keller HH, Rysdale LA, Beyers JE (2008). Nutrition Screening Tool for Every Preschooler (NutriSTEP): validation and test-retest reliability of a parent-administered questionnaire assessing nutrition risk of preschoolers. Eur J Clin Nutr.

[67] Benjamin S, Neelon B, Ball S, Bangdiwala S, Ammerman A, Ward D (2007). Reliability and validity of a nutrition and physical activity environmental self-assessment for child care. Int J Behav Nutr Phys Act.

[68] Benjamin S, Ammerman A, Sommers J, Dodds J, Neelon B, Ward D (2007). Nutrition and physical activity self-assessment for child care (NAP SACC): results from a pilot intervention. J Nutr Educ Behav.

[69] Trost S, Messner L, Fitzgerald K, Roths B (2009). Nutrition and physical activity policies and practices in family child care homes. Am J Prev Med.

[70] Ward D, Morris E, McWilliams C, Vaughn A, Erinosho T, Mazzuca S, Hanson P, Ammerman A, Neelon S, Sommers S, Ball S (2013). Go NAP SACC: Nutrition and Physical Activity Self-Assessment for Child Care.

[71] Bandura A (1986). The explanatory and predictive scope of self-efficacy theory. J Soc Clin Psychol..

[72] Bandura A, McClelland D (1977). Social learning theory.

[73] Lee P, Macfarlane D, Lam T, Stewart S (2011). Validity of the international physical activity questionnaire short form (IPAQ-SF): A systematic review. Int J Behav Nutr Phys Act.

[74] Canada S (2010). Canadian Community Health Survey (CCHS).

[75] Martinent G, Naisseh M, Ferrand C, Bois J, Hautier C (2013). Development and evaluation of the psychometric properties of the parents’ Perceptions of Physical Activity Importance and their Children’s Ability Questionnaire (PPPAICAQ). Psychol Sport Exerc.

[76] National Health and Nutrition Examination Survey. Anthropometry Procedures Manual. 2004. p. 1-65.

[77] Monasta L, Lobstein T, Cole T, Vignerová J, Cattaneo A (2011). Defining overweight and obesity in pre-school children: IOTF reference or WHO standard?. Obes Rev.

